# Spin-Orbit Effects on the Dynamical Properties of Polarons in Graphene Nanoribbons

**DOI:** 10.1038/s41598-018-19893-y

**Published:** 2018-01-30

**Authors:** Luiz Antônio Ribeiro, Gesiel Gomes da Silva, Rafael Timóteo de Sousa, Antonio Luciano de Almeida Fonseca, Wiliam Ferreira da Cunha, Geraldo Magela e Silva

**Affiliations:** 1 Department of Physics, Chemistry and Biology (IFM), Linköping University, SE-581 83 Linköping, Sweden; 2 Institute of Physics, University of Brasίlia, 70.919-970 Brasίlia, Brazil; 3 Goias Federal Institute of Science and Technology, IFG, Luziânia, 72.811-580 Brazil; 4 Department of Electrical Engineering, University of Brasίlia, Brasίlia, 70919-970 Brazil

## Abstract

The dynamical properties of polarons in armchair graphene nanoribbons (GNR) is numerically investigated in the framework of a two-dimensional tight-binding model that considers spin-orbit (SO) coupling and electron-lattice (e-l) interactions. Within this physical picture, novel polaron properties with no counterparts to results obtained from conventional tight-binding models are obtained. Our findings show that, depending on the system’s width, the presence of SO coupling changes the polaron’s charge localization giving rise to different degrees of stability for the charge carrier. For instance, the joint action of SO coupling and e-l interactions could promote a slight increase on the charge concentration in the center of the lattice deformation associated to the polaron. As a straightforward consequence, this process of increasing stability would lead to a depreciation in the polaron’s motion by decreasing its saturation velocity. Our finds are in good agreement with recent experimental investigations for the charge localization in GNR, mostly when it comes to the influence of SO coupling. Moreover, the contributions reported here provide a reliable method for future works to evaluate spin-orbit influence on the performance of graphene nanoribbons.

## Introduction

Graphene-based optoelectronic devices have emerged, in the past decade, as promising solutions in developing novel technologies for green energy applications of considerably low environmental impact^1–4^. Most of the interesting properties of a graphene sheet come from its flexible two-dimensional structure (a hexagonal lattice), which leads this material to present singular features such as excellent heat and electrical conductivity^5,6^, high mechanical strength^7^, and remarkable optical properties, including good transparency for visible light^8^. Graphene Nanoribbons (GNR), in turn, are derived from specific cuts of the graphene sheet^9^. Interestingly – depending on the way of cutting – they may preserve some properties of the original system and present a finite band gap^10,11^. In a GNR, the most important trait in developing new optoelectronic applications is its semiconducting-like electronic structure^12–14^. It is well known that in organic semiconducting materials the charge transport mechanism is mediated by polarons^15,16^. Therefore, a detailed knowledge about the dynamical properties of these charge carriers in GNR may open new channels for improving the performance of devices in which graphene-based materials play an important role.

Recently, some relevant experimental results have investigated the electronic structure of graphene edges^17^ and nanoribbons^18^ with particular interest at describing the charge localization signatures. Yang and coworkers have studied the quantum interferences at different edge structures – irregular armchair, mixed armchair and zigzag, and regular armchair – of a graphene sheet by using atomic-scale scanning tunneling microscopy (STM) topographies^17^. They have observed that quantum interferences form high electronic density of states patterns along the carbon-carbon bonds, whose shapes depend strictly on the edge structure and not on the electron energy. Also by means of STM measurements, Huang and colleagues have investigated the electronic structure of armchair GNR (AGNR) upon deposition on silver substrates^18^. Their results revealed one-dimensional delocalized striped patterns for the electronic density of states in such a way that the electronic charge is distributed along the nanoribbon length. The above-mentioned studies provide a very nice physical picture about the overall trend of charge localization in GNR. Nevertheless, the dynamical properties of charge carriers (polarons), mostly when it comes to the role played by the presence of a spin-orbit coupling (SOC) term, is not completely understood and requires a more detailed investigation.

In this work, a systematic numerical study is employed to investigate the influence of SOC interactions on the dynamical properties of polarons in AGNR using a two-dimensional tight-binding approach that includes lattice relaxation effects. The usefulness of our model is highlighted by studying the polaron’s properties of different AGNR families. The present study is aimed to provide a deep physical understanding about the configuration of charge carriers in GNR and, consequently, the impact of these properties on the charge transport mechanism in these systems.

## Methodology

The two-dimensional tight-binding model considered here consists of a modified version of the Su-Schrieffer-Heeger Hamiltonian^19,20^. In this way, the overall Hamiltonian (*H*) has the form: *H* = *H*_*elec*_ + *H*_*latt*_ + *H*_*so*_, where *H*_*elec*_, *H*_*latt*_, and *H*_*so*_ address the electronic part, the lattice backbone, and the spin-orbit coupling term, respectively. The electronic contribution to the total Hamiltonian can be placed as

1 $${H}_{elec}=-\sum _{\langle i,j\rangle ,s}[{e}^{-i\gamma {A}_{i,j}}({t}_{0}-\alpha {y}_{i,j}){C}_{i,s}^{\dagger }{C}_{j,s}+h\mathrm{.}c\mathrm{.}]\mathrm{.}$$

In the expression above, *i* and *j* label two arbitrary neighboring sites in the lattice and *y*_*i*,*j*_ denotes the variation in the bond length for two such sites. ${C}_{i,s}^{\dagger }$ (*C*_*j*,*s*_) creates (annihilates) a π electron with spin *s* in the $i-th$ ($j-th$) site. *t*_0_ is the transfer integral in a pristine lattice in which the atoms are equally spaced and *α* is the electron-lattice coupling constant that takes into account the interplay between the two distinct degrees of freedom defined by the electronic and lattice contributions. $\gamma \equiv ea/(\hslash c)$ where *e* is the electric charge, *a* the lattice constant, and *c* the speed of light. The external electric field is included by using the time-dependent vector potential **A(t)**, and is derived as ${\bf{E}}({\bf{t}})=-(1/c){\bf{A}}({\bf{t}})$^21^.

The lattice backbone dynamics, in its turn, is addressed in a harmonic approximation – which accounts the effective potential associated with the *σ* bonds – in the following form

2 $${H}_{latt}=\frac{1}{2}\sum _{\langle i,j\rangle }K{({y}_{i,j})}^{2}+\frac{1}{2}\sum _{i}\frac{{P}_{i}^{2}}{M}\mathrm{.}$$

In this expression, *K* is the force constant, *P*_*i*_ is the conjugated momentum of the *i* − *th* site, and *M* the site mass. The last term in the total Hamiltonian considers the contribution of the SO interactions and can be written as

3 $${H}_{so}=i\sum _{\langle j,l\rangle ,s,s^{\prime} }{t}_{so}{\zeta }_{j,l}{C}_{j,s}^{\dagger }{s}_{z}{C}_{l,s^{\prime} }$$

where the index *l* goes through all the six next-nearest-neighbors of the atom *j*. The parameter ${\zeta }_{j,l}=1(-1)$ for anti-clockwise (clockwise) spin-orbit interaction if *j* is in sublattice *A*, and vice versa if *j* is in sublattice *B*^22^. *s*_*z*_ is the Pauli matrix and *t*_*so*_ is the spin-orbit hopping term. In our simulations, *t*_*so*_ is settled to be a tenth of *t*_0_^22^.

The initial arrangement of the system contains a positive polaron in its ground state configuration. Such situation is achieved by using the self-consistent procedure described in ref.^23^. The time evolution of the system is governed by an Ehrenfest Molecular Dynamics approach, according to ref.^23^. The parameters adopted here for the model Hamiltonian were successfully employed in other works presenting a good track-record^23–28^: *t*_0_ = 2.7 eV, *M* is the carbon core’s mass, *K* = 21 eV/Å^2^, *α* = 4.1 eV/Å, and *a* = 1.44 Å.

## Results

**Figure 1:**
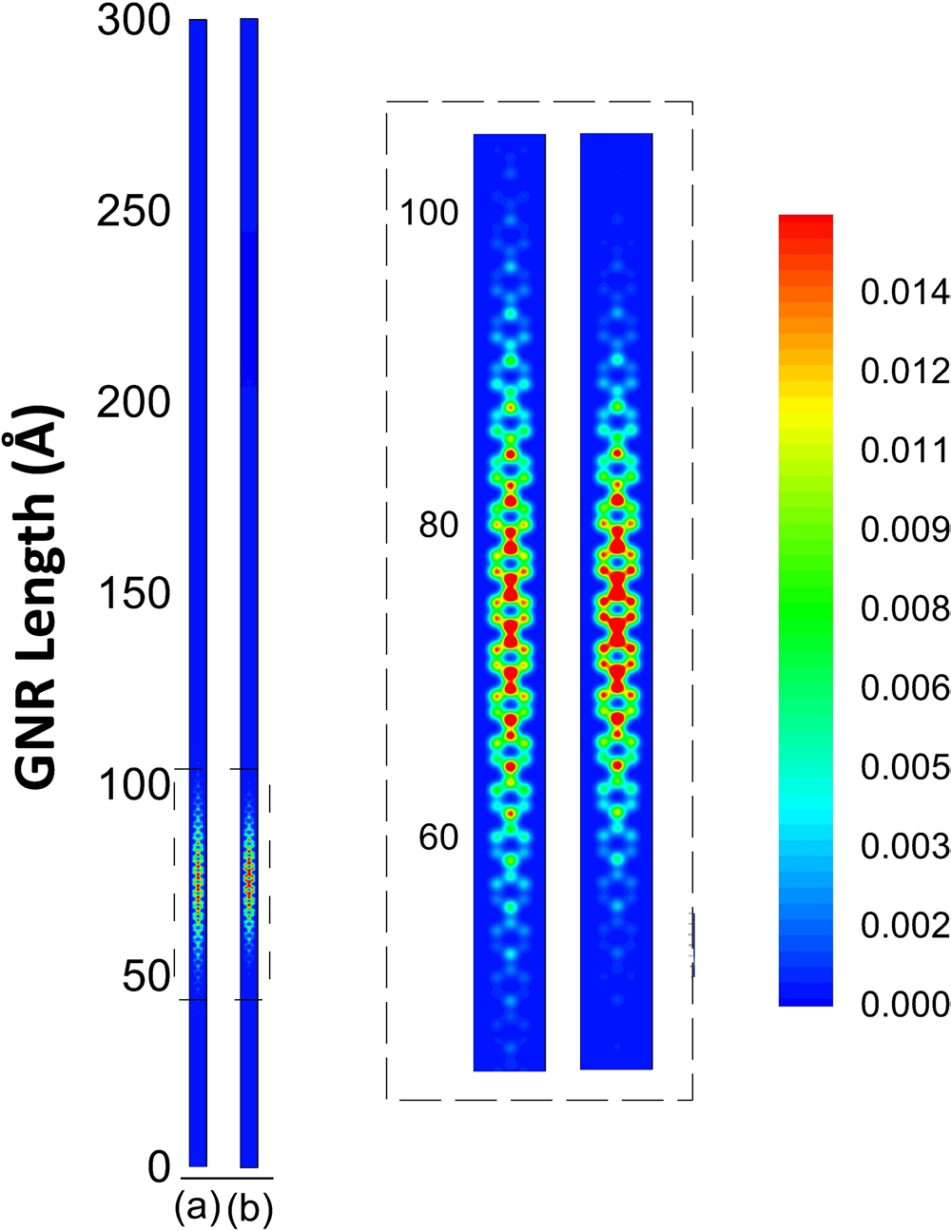
Comparison between the charge distribution of AGNR with 3 atoms width (**a**) without and (**b**) with SO coupling.

We begin our discussion by presenting the impact of SO interactions on the ground state properties of polarons, with particular interest on its charge localization pattern. In this way, Fig. 1 presents the comparison between the charge distribution of AGNR with 3 atoms width without (a) and with (b) SO coupling. This is carried out by plotting the order parameter for the charge distribution (as described in^24^) when the extraction of a single electron was carried out, using the same adiabatic procedure in both cases. The SO parameter was set to be a tenth of the transfer integral parameter *t*_0_, as previously reported in the literature^22^. Because the nanoribbon from this figure is a representative of the 3p family, which is known to be of semiconducting nature^10,11^, in both cases we observe a localization of the charge carrier. This localization is associated with a quasi-particle mediated mechanism for this kind of chain. The most striking feature that can be observed from this comparison is the higher localization of the charge in the SO endowed model. This property follows from the larger coupling between the system’s components that the SO parameter introduces to the system.

**Figure 2:**
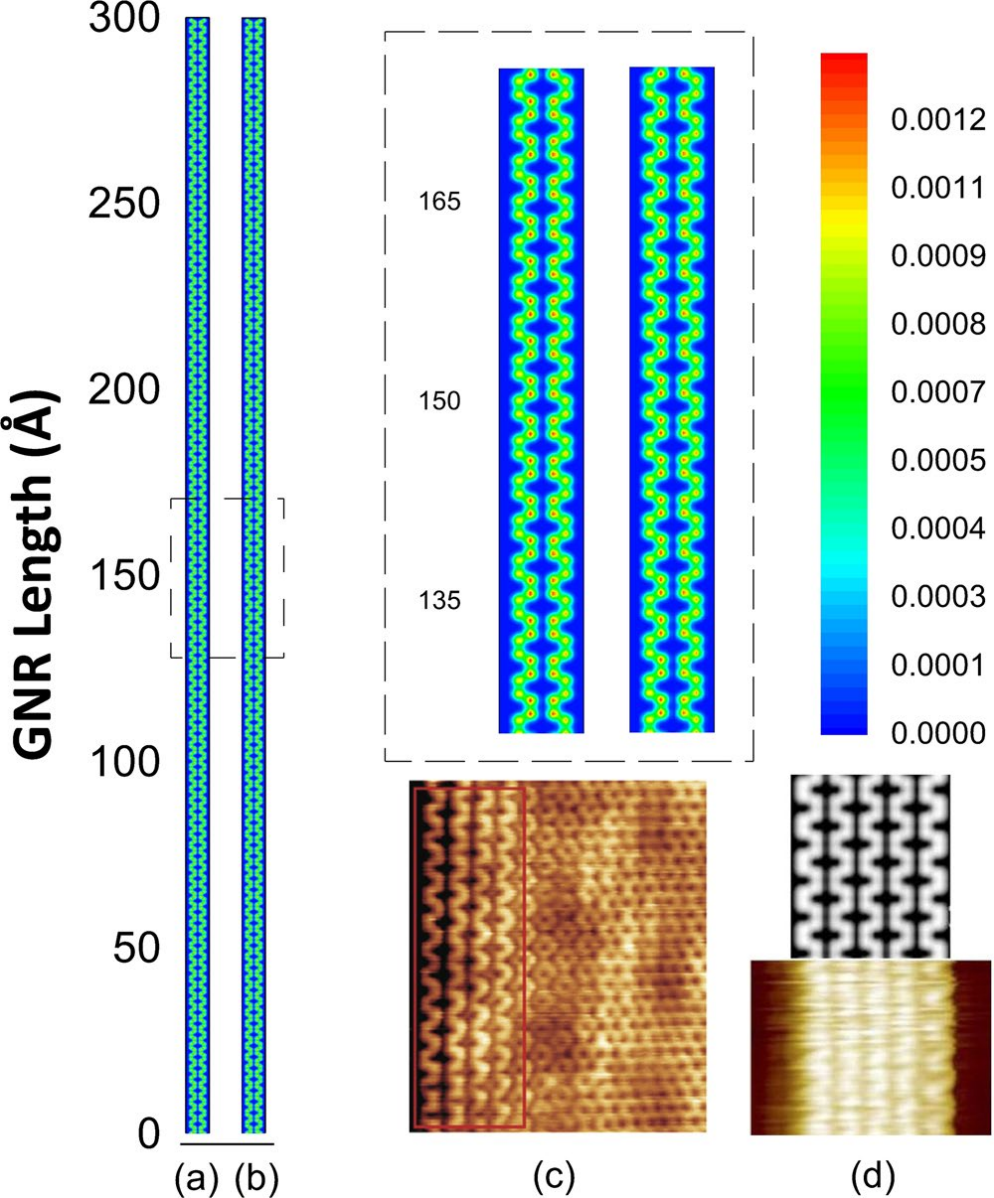
Comparison between the charge distribution of AGNR with 5 atoms width (**a**) without and (**b**) with SO coupling. The panels (c) and (d) were reproduced from the references^17^ and^18^, respectively.

One could be interested to investigate the role played by this parameter in armchair nanoribbons of the 3p + 2 family, which is known to present zero gap. Figure 2 presents, for these metallic nanoribbons, a comparison similar to the one carried out in Fig. 2. One can see that, although the delocalization of the charge is similar, the charge in AGNR-5 is completely delocalized through the lattice. Besides the difference in the charge localization, one can also note that, in this case, charge is symmetrically displaced throughout all the ribbon’s length regardless of considering SO coupling or not. As expected, this behavior is the one observed both by experimental measurements of scanning tunneling microscopy^17^ (Fig. 2(c) and bottom image of Fig. 2(d)) and *ab-initio* theoretical calculations in the framework of Density Functional Theory^18^ (top image of Fig. 2(d)). As our goal is to investigate charge carrier mediated mechanism, we will focus in nanoribbons of the semiconducting families only, i.e., 3p and 3p + 1.

**Figure 3:**
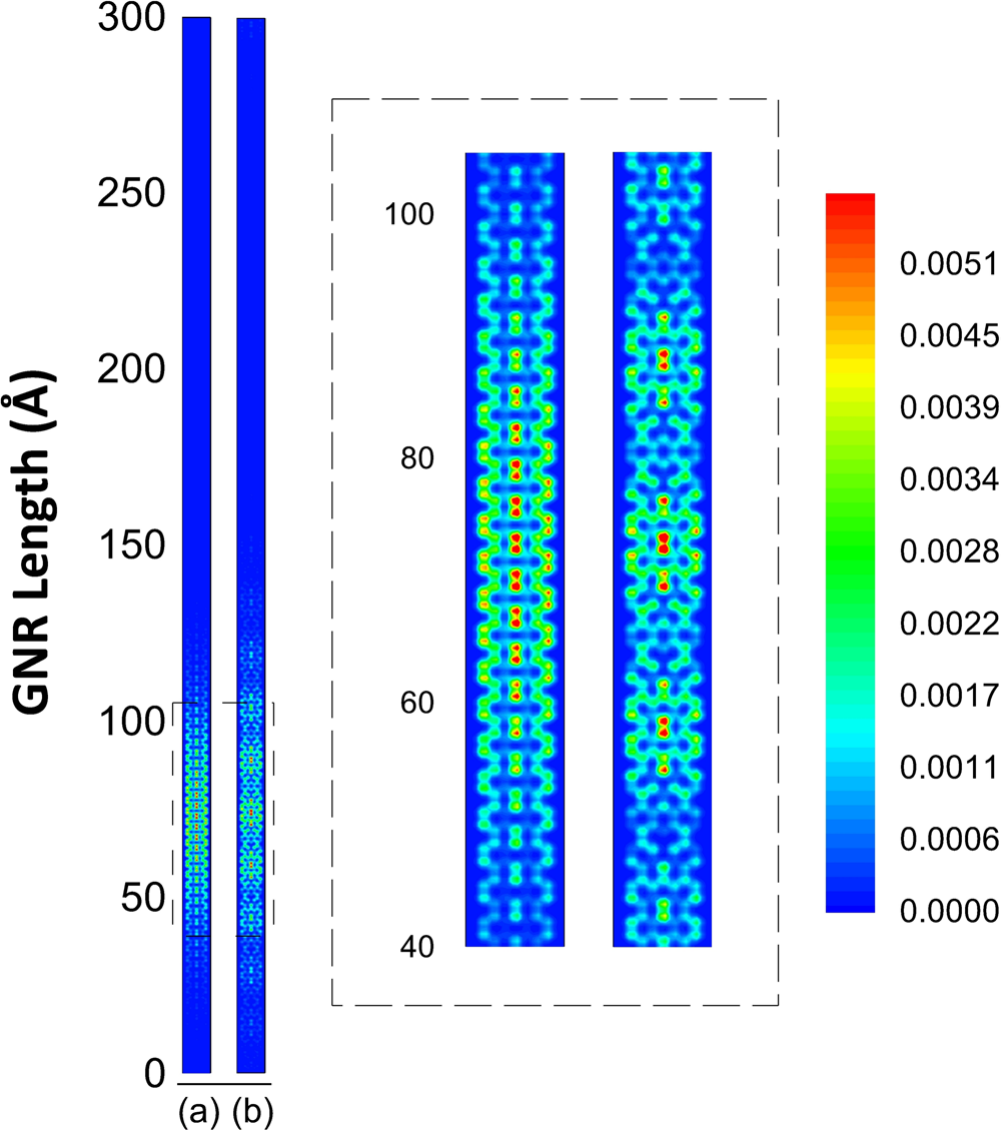
Comparison between the charge distribution of AGNR with 7 atoms width (**a**) without and (**b**) with SO coupling.

By further increasing the width size of the investigated semiconducting nanoribbon from 3 to 7 we reach a representative of a 3p + 1 family in which an interesting effect of SO coupling can be observed. From Fig. 3, one can see that the very nature of the distribution is strongly affected by the inclusion of the SO parameter. In Fig. 3(a), when no SO coupling is present, one can see that the distribution follows the same trend of Fig. 1. Actually, the differences observed are due to the fact that the nanoribbon is broader so the charge is more distributed along its width. One can note three centers of charges (one above each carbon backbone) and also a smooth decrease of charge concentration from the central position. Figure 3(b), on the other hand, presents a rather different behavior: the charge is symmetrically displaced in quasi-localized centers of charge. Three such centers can be clearly distinguished in the figure. Each one of these centers presents its charge relatively more concentrated than the mean concentration of charge in the absence of SO coupling. This, again, is closely related to the fact that SO coupling tends to introduce an increase of charge localization. This aspect has important consequences and will be further explored later.

**Figure 4:**
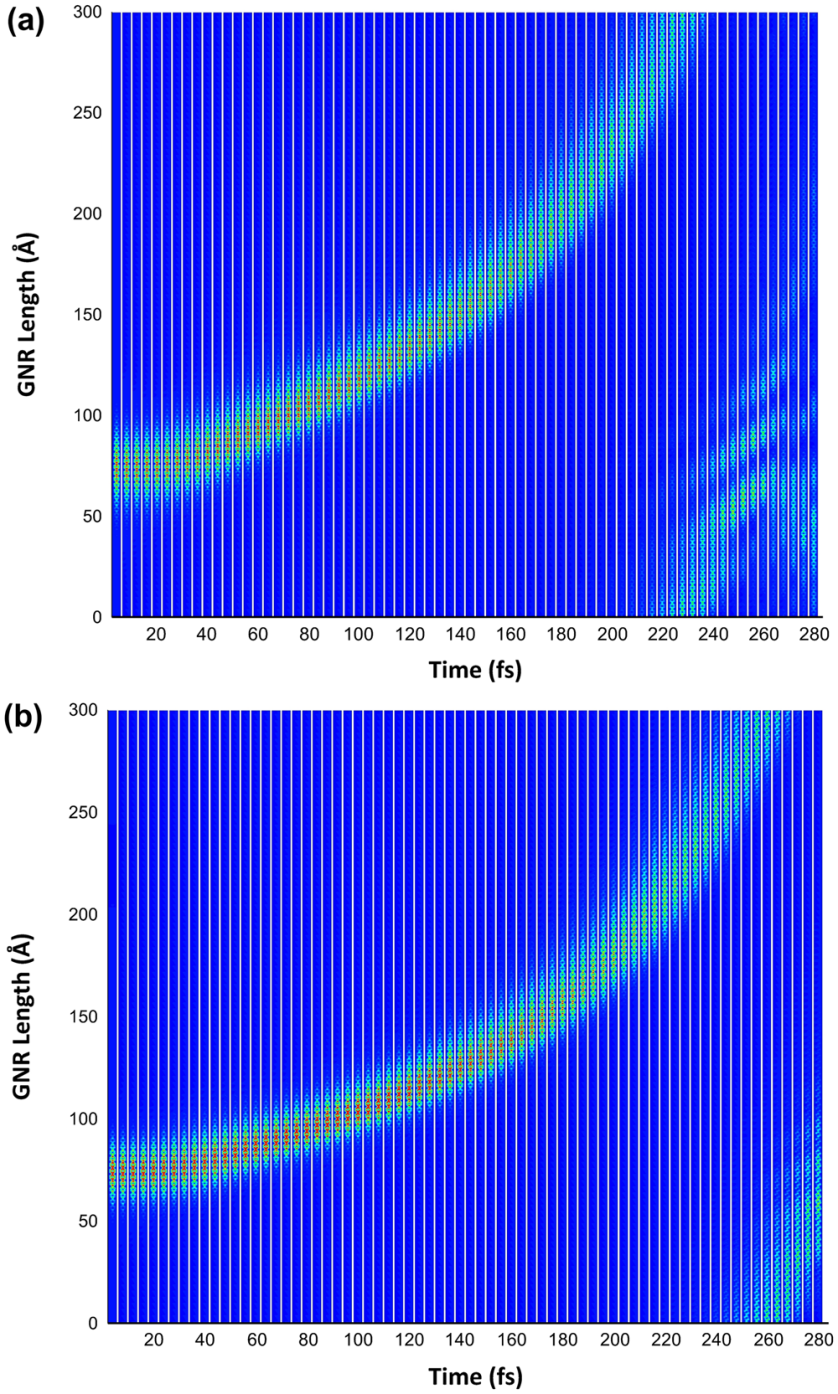
Comparison between the dynamics of the charge carriers at AGNR with 3 atoms width (**a**) without and (**b**) with SO coupling.

Now, we turn our attention to the comparison between the dynamics of the charge carriers with and without SO coupling for the AGNR with 3 and 7 atoms width. Note that, in order to restrict edge effects, periodic boundary conditions were considered in the nanoribbon’s length, just as in ref.^23^. The electric field strength is settled to be 1.5 mV/Å for all cases and is oriented along the nanoribbon’s length. The former is discussed in Fig. 4. The electric field value was chosen according to what was previously reported in the literature^26^. The general behavior of the charge carrier transport is very similar in Fig. 4(a), in which no SO parameter was considered, and 4(b), where we present the results of such effect. In both figures, the charge carrier discussed in Fig. 1 responds to the applied electric field. These results were obtained by the same procedure explained in ref.^24^. Note that the response time, which is roughly the same in both cases, is due to the fact that the field was adiabatically introduced in the system. After a simulated time of approximately 30 fs, the carrier follows the direction of the applied field and its dynamics is consistent to what was previously reported in the literature^23,26^. The importance of the SO consideration can be better appreciated by the comparison between the two situations, rather than in the description of the process itself. The first interesting feature that can be noted is that the higher localization degree implemented by the inclusion of SO effects – which has been already pointed out in Fig. 1 – remains consistent through all the simulation. In other words, the electric field and the dynamic process itself do not destabilize the carrier. We conclude that such concentration effect is a manifestation of a different nature in the transporting polaron. In other words, SO effect gives rise to a polaron of a different nature when compared to simulations with uncoupled degrees of freedom. As a straightforward consequence, one can see that the more concentrated polaron presents a smaller average speed. By taking the periodic boundary condition as reference, it is easy to note that, whereas the spin-orbit coupled polaron takes around 260 fs to span the 225 ${\rm{\AA }}$ of the chain, the one with no SO parameter takes approximately 220 fs. This is a direct result of the localization degree: the more delocalized the charge carrier, the higher its mobility, a fact that has been extensively explored in the literature^29–32^.

**Figure 5:**
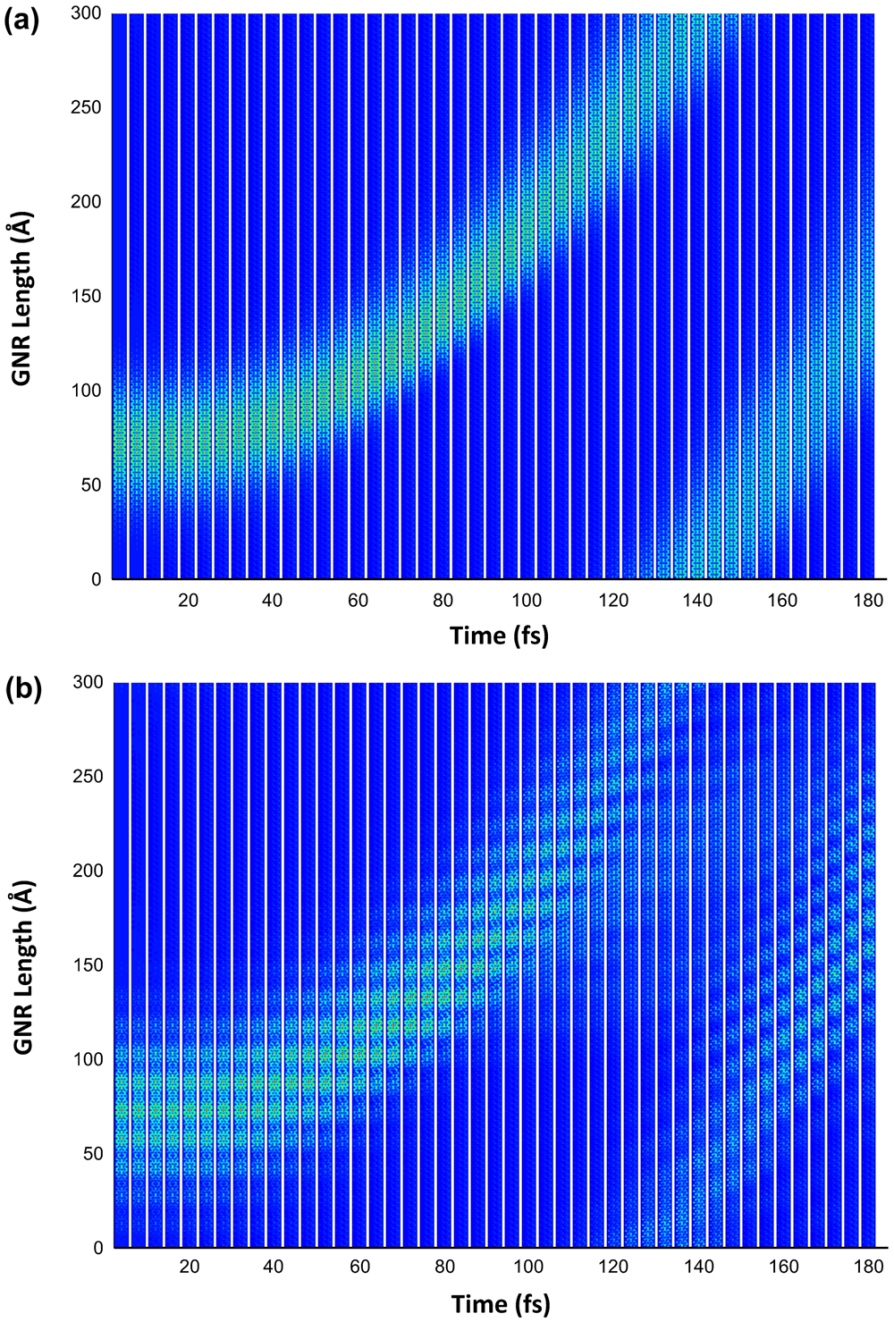
Comparison between the dynamics of the charge carriers at AGNR with 7 atoms width (**a**) without and (**b**) with SO coupling.

An interesting issue arises on whether the higher localization – and thus the smaller mobility – is an inexorable consequence of the inclusion of SO coupling. In order to verify this possibility, we investigate the dynamics of charge carriers on an AGNR-7 chain, for which we have seen that the initial static solution is highly dependent on considering or not such coupling. We have seen that the SO effect plays the role of creating a completely different distribution pattern for the polaron as the initial state of the system. Figure 5 presents the time evolution of the charge carrier under the influence of electric field. One can see that the charge distribution profile of both the polaron in a SO uncoupled system (Fig. 5(a)) and of the polaron in the presence of the coupling parameter (Fig. 5(b)) remains stable throughout the simulation, which is consistent to the previous case. What is observed in this case, however, is that even though Fig. 5(b) is associated with the inclusion of SO coupling, its average velocity is not larger than that of Fig. 5(a), where this effect was not considered. This is related to the fact that, although the charge is differently displaced in the two situations, the delocalization length is roughly the same.

**Figure 6:**
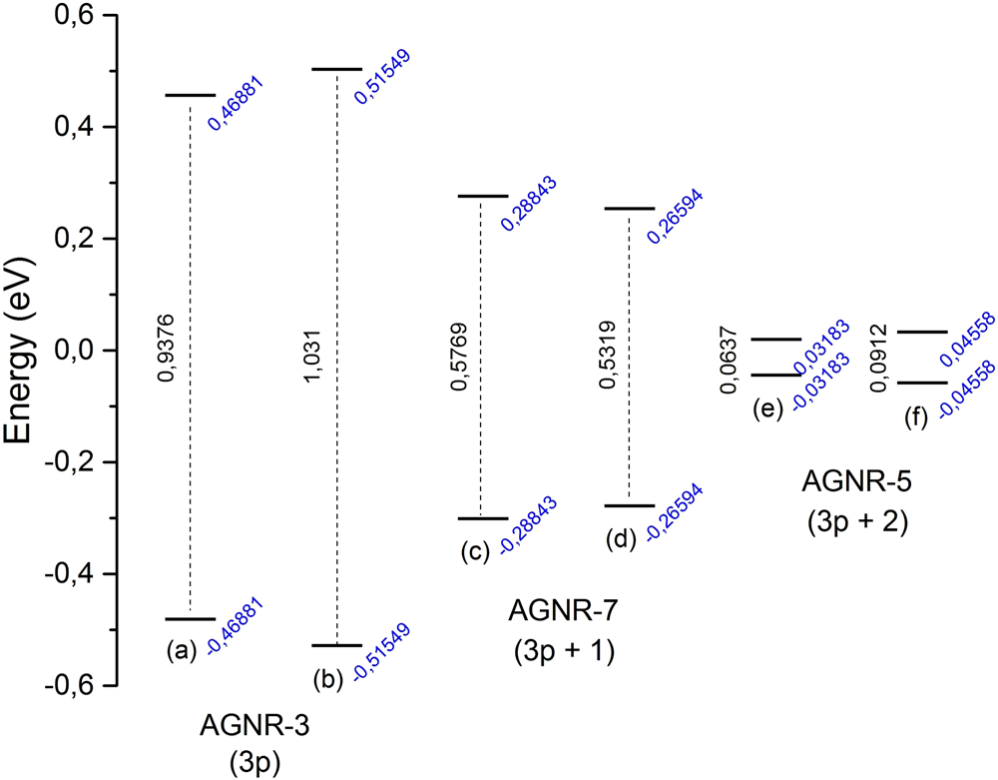
Comparison between the HOMO-LUMO Energy Gaps for all the systems studied in our work. HOMO refers to the Highest Occupied Molecular Orbital whereas LUMO refers to the Lowest Unoccupied Molecular Orbital.

The comparison between the result of Fig. 4 and that of Fig. 5 comprises the main finding of the present paper: the inclusion of SO coupling has the effect of changing the overall nature of the polaron. This does not mean necessarily that the distribution length or shape of the polaron will be altered in a given sense: the nature of the change is dependent on the size and the family of the nanoribbon, for the geometry change affects the coupling between charge and spin differently. However, this coupling will inevitably conduct to a change in some property of the carrier to the extent that we can attribute a rather different nature of the charge carrier. We conclude our analysis on the different nature of the polarons of SO coupled endowed systems by studying the HOMO-LUMO energy gap for all the systems studied in our work. Figure 6 presents the comparison of the situation without and with SO parameters for the three families. Note that, as mentioned above, there is always an expected change of the energy gap, but the direction in which this change is observed depends on the family of the nanoribbon. This change is a manifestation of the change in the nature of the polaron. Note that our findings should have profound influence on the literature, since most dynamic works performed so far disregarded SO effects in graphene nanoribbons.

## Conclusions

In summary, the ground state and dynamical properties of polarons for different families of AGNR were numerically studied in the scope of a two-dimensional tight-binding model that considers SO coupling and electron-lattice interactions. The results show that there is a higher localization of the polaron when a SO term is taken into account. This is accomplished by the larger coupling between the system’s components (charge and lattice), which is introduced by including the SO parameter. Importantly, it was demonstrated that – besides the difference in the charge localization – for AGNR belonging to 3p + 2 family the electronic charge is symmetrically displaced throughout all the ribbon’s length regardless of considering SO coupling or not. These findings agree with both theoretical and experimental results recently reported in literature^17,18,28^. Regarding the polaron dynamics, it was obtained that the electric field and the dynamic process itself do not destabilize the carrier in the presence of a SO coupling term. Therefore, one can conclude that the concentration effect (promoted by the SO coupling term) is a manifestation of a different nature in the transporting polaron. More clearly, the SO effect gives rise to a polaron of a different nature when compared to simulations with uncoupled degrees of freedom. As a natural consequence, one can realize that the more concentrated polaron presents a smaller average speed.
